# Identifying Immunological Biomarkers for Major Depressive Disorder: Insights From Machine Learning, Single‐Nucleus Bioinformatics, and Experimental Validation

**DOI:** 10.1155/bmri/6184295

**Published:** 2026-04-11

**Authors:** Long Kangsheng, Yang Xiaohui, Pei Xin, Ye Yong, Li Hongliang, Deng Yihui

**Affiliations:** ^1^ The First Affiliated Hospital of Hunan University of Chinese Medicine, Changsha, Hunan, China, hnctcm.edu.cn; ^2^ Hunan University of Chinese Medicine, Changsha, China, hnctcm.edu.cn

**Keywords:** bioinformatics, biomarkers, machine learning, major depressive disorder, single-nucleus RNA sequencing

## Abstract

**Background:**

Major depressive disorder (MDD) is a chronic mental illness rapidly approaching the status of a significant global burden of disease. This study is aimed at investigating novel biomarkers in MDD and performing a comprehensive analysis of immune infiltration through an integrated bioinformatics approach.

**Methods:**

The study involved differential gene expression analysis, weighted gene coexpression network analysis (WGCNA), single‐nucleus RNA sequencing (snRNA‐seq), and the application of three machine learning methods. Additionally, we constructed the chronic unpredictable mild stress (CUMS) rat model of MDD, and their brain tissues were analyzed by transcriptional sequencing to explore the differentially expressed genes (DEGs). Finally, quantitative reverse transcription–polymerase chain reaction (RT‐qPCR) and western blot experiments were conducted to verify the expression level of hub gene in brain tissues.

**Results:**

A total of 132 DEGs were discovered, with enrichment analysis revealing their significant involvement in immune‐related functions and pathways. WGCNA analysis yielded three hub genes (DACH1, FZD7, and GULP1). These hub genes were identified by intersecting candidate signature genes obtained from three machine learning analyses with DEGs. SnRNA‐seq analysis revealed significant differences in immune cell–related expression patterns between MDD patients and healthy controls. The presence of three hub genes was found by DEGs in brain tissues of CUMS rats. Further RT‐qPCR and western blot experiments demonstrated that DACH1 and GULP1 were upregulated, and FZD7 was downregulated in brain tissues of CUMS rats.

**Conclusion:**

Our findings contribute to the understanding of the relationship between MDD and immune infiltration. DACH1, FZD7, and GULP1 may be key biomarkers and potential therapeutic targets for MDD.

## 1. Introduction

Major depressive disorder (MDD) is a chronic mental health condition characterized by persistent symptoms, including a depressive mood, emotional distress, functional impairment, health issues, and an elevated risk of suicide [1]. In the United States, the 12‐month prevalence rate for this psychiatric disorder is 10.4%, with a lifetime prevalence rate of 20.6% [2] [3]. Various risk factors contribute to MDD, such as childhood adversity, stress, despair, and aggression, potentially leading to suicidal ideation and behavior [4]. Mental illness, especially MDD, plays a significant role in more than 85% of suicide cases, emphasizing the critical importance of mental health in suicide prevention [5].

Despite extensive genome‐wide association studies, the neuropathological mechanisms of MDD remain unclear, creating challenges for accurate diagnosis and effective treatment. Gene expression analysis has identified numerous genes and disease‐related information associated with MDD [6, 7]. A large‐scale meta‐analysis, incorporating data from over 800,000 individuals, successfully identified 102 independent variants, 269 genes, and 15 gene sets related to depression, all showing significant genome‐wide associations [8]. However, depression is a heterogeneous syndrome, and our understanding of its pathophysiology and pathogenesis is still limited. Recent studies indicate a connection between the onset and progression of MDD and the activation of the immune system [9]. However, current comprehension of the pathogenesis of MDD remains constrained. Recognizing the diversity of cell types and their intricate interactions in the brain, there is a clear need for cell type–specific investigation methods to gain a deeper understanding of psychiatric phenotypes.

Bioinformatics is an emerging science and technology for analyzing gene expression, which helps to reveal potential key genes and key pathways and provides technical support for analyzing the molecular mechanisms of the disease [10]. Single‐cell sequencing allows for the identification of cellular sequence differences in specific microenvironments to study functional distinctions, whereas RNA sequencing aids in understanding and identifying various cell types along with their expressed genes [11]. Recent developments in single‐nucleus RNA sequencing (snRNA‐seq) have significantly enhanced sensitivity, accuracy, and efficiency [12]. In comparison with single‐cell RNA sequencing (scRNA‐seq), snRNA‐seq offers notable advantages in effectively isolating individual cell nuclei, particularly in handling frozen tissue, where it excels. Through the separation of organizational collection and real‐time sample processing, this method can proficiently manage fresh samples that might pose challenges for separation [13].

In this study, we initially utilized MDD‐associated microarray profiles from the Gene Expression Omnibus (GEO) dataset. We delved into the analysis of differentially expressed genes (DEGs) between the MDD and control groups. Employing three machine learning techniques, we are aimed at identifying potential diagnostic biomarkers for MDD. Additionally, we explored the association between the identified biomarkers and infiltrating immune cells. To gain a clearer understanding of the mechanisms involved in disease progression, we leveraged snRNA‐seq technology to scrutinize these biomarkers at the cellular level. Finally, we performed transcriptome sequencing on brain tissues from normal rats and chronic unpredictable mild stress (CUMS) rats of the MDD model to further validate the presence of these immune‐related hub genes. RT‐PCR and western blot were performed to verify the expression differences of these immune‐related hub genes between the CMUS group and the control group. Through a series of bioinformatics analyses, the link between MDD and immunity was identified, and the potential diagnostic value of these immune‐related hub genes and their relevance to MDD pathogenesis were further clarified, which hold promise for advancing diagnostic and therapeutic strategies for MDD.

## 2. Materials and Methods

### 2.1. Data Acquisition

Publicly available transcriptomic datasets related to MDD were retrieved from the GEO database (https://www.ncbi.nlm.nih.gov/geo/). Dataset selection was based on the following criteria: inclusion of postmortem brain tissue samples from both MDD patients and nonpsychiatric controls, availability of complete expression matrices and corresponding platform annotation files, and sufficient sample size and basic clinical metadata to allow downstream comparative analyses.

Based on these criteria, GSE53987 (controls = 55, MDD = 50) and GSE92538 (controls = 56, MDD = 29) were selected as the primary datasets for differential gene expression analysis. Raw expression data were processed using platform‐specific annotation files, and probe IDs were mapped to gene symbols using Perl scripts, retaining only uniquely annotated genes. To minimize systematic technical variation arising from differences in sample processing and platform effects, batch correction was performed using the ComBat function implemented in the “sva” R package.

**Figure 1:**
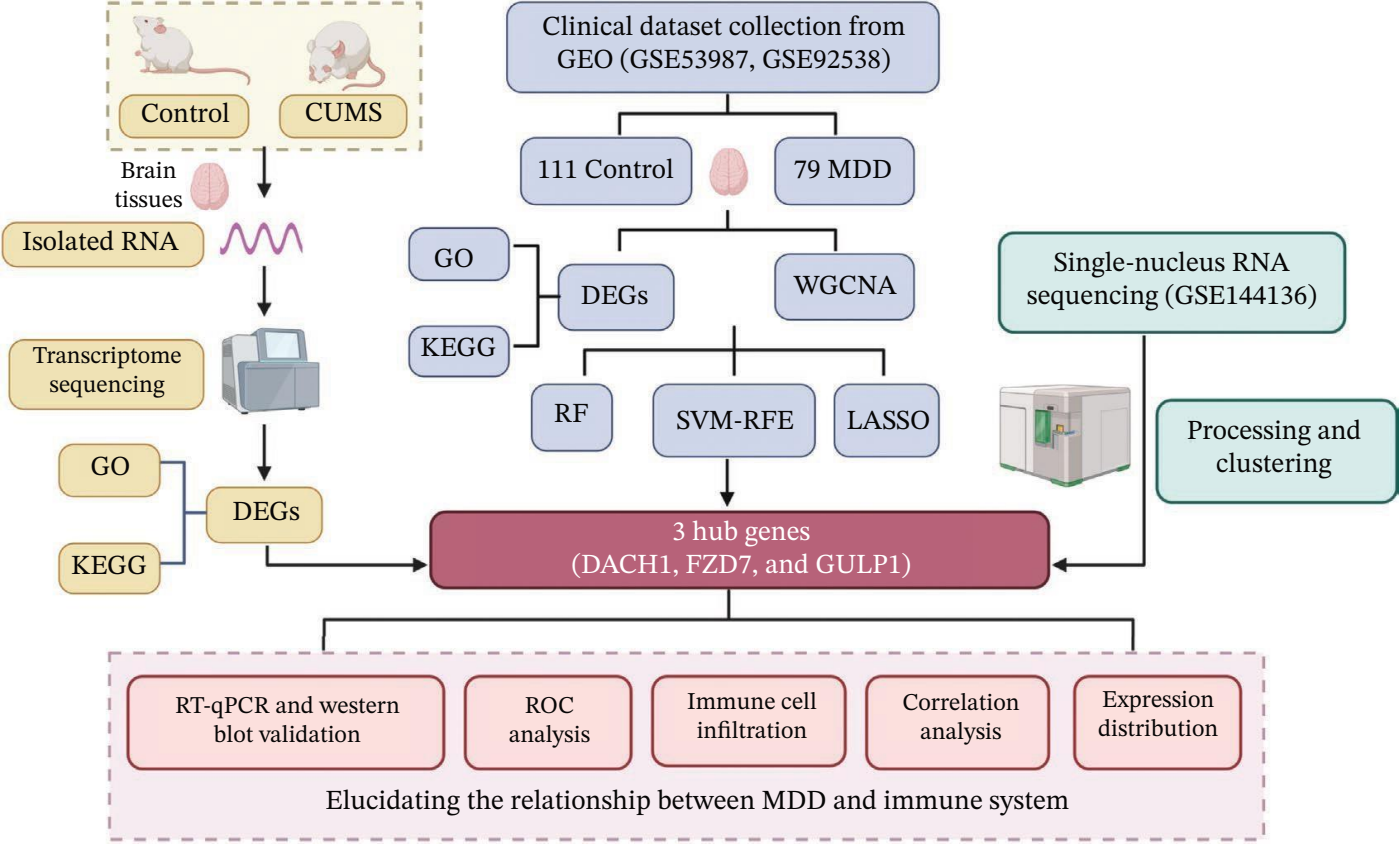
The workflow of the study.

GSE54568 (GPL570; 15 MDD cases and 15 controls) was designated as an independent test dataset to validate characteristic genes identified in the training cohort. In addition, GSE144136 (GPL20301), which provides complete expression profiles from MDD and control samples, was incorporated for downstream snRNA‐seq analysis to further explore cell type–specific expression patterns. The overall workflow, dataset selection strategy, and analytical procedures are illustrated in Figure 1.

Clinical characteristics of MDD patients and control subjects included in the differential expression analysis are summarized in Table S1. No statistically significant differences were observed between the two groups with respect to age, sex, postmortem interval (PMI), or tissue pH, suggesting an overall comparability of baseline demographic and postmortem variables between groups.

### 2.2. Shortlisting of MDD‐Related Genes and WGCNA Analysis

Differential expression analysis between MDD and control samples was performed using the “limma” R package. Genes with |log_2_ fold change (FC)| > 0.5 and *p* < 0.05 were defined as DEGs. Heatmaps and volcano plots were generated using the “pheatmap” and “ggplot2” packages, respectively.

WGCNA was conducted on the DEG set (*n* = 132) to identify coexpression modules associated with MDD. The soft‐thresholding power was determined using the *pick soft threshold* function by evaluating scale‐free topology fit and mean connectivity across candidate powers (*β* = 1–20), and *β* = 17 was selected. Pearson correlation was used to construct the adjacency matrix and topological overlap matrix (TOM). Gene modules were identified by hierarchical clustering with dynamic tree cutting (minModuleSize = 50), followed by module merging based on eigengene similarity. Module–trait associations were assessed by correlating module eigengenes with the MDD phenotype using Pearson correlation analysis.

### 2.3. Functional Enrichment and Identification of Critical Gene

Conducting Gene Ontology (GO) functional annotation and Kyoto Encyclopedia of Genes and Genomes (KEGG) pathway analysis is aimed at deepening our understanding of the biological functions of MDD and related signaling pathways [14]. Visualization of functional enrichment and pathway analysis was carried out using the “GOplot” and “ggplot2” software packages in R, respectively [15].

Three machine learning algorithms were employed to identify crucial markers for predicting MDD. Initially, the “glmnet” software package was utilized to set alpha = 1 and perform 10‐fold cross‐validation to adjust parameter selection, conducting least absolute shrinkage and selection operator (LASSO) regression analysis on candidate genes. The application of support vector machine (SVM) in disease genomics learning aids in discovering new biomarkers and understanding disease‐driving genes. Recursive feature elimination (RFE) selects the optimal combination of features by eliminating redundancy between them [16]. Finally, genes generated through LASSO, SVM‐RFE, and RF algorithms were considered as characteristic biomarkers for diagnosing MDD. Violin and heatmaps were used to display the target gene levels of the MDD group and the normal healthy group.

To evaluate the clinical utility and diagnostic performance of the characteristic genes for MDD, receiver operating characteristic (ROC) curves were generated using mRNA expression datasets from GSE54568 (GPL570) and GSE87610 (GPL13667) [17].The area under the ROC curve (AUC) was calculated to assess predictive accuracy. In addition, decision curve analysis (DCA) was performed using the “rmda” R package to evaluate the net clinical benefit of the diagnostic model across a range of threshold probabilities. DCA provides complementary evidence beyond AUC by quantifying the potential clinical value of using the gene‐based model for decision‐making.

### 2.4. Nomogram Construction

The nomogram, constructed through multiple regression analysis, integrates various predictive indicators. Employing scaled line segments, it visually represents the interrelationships between variables in the predictive model. In this study, the nomogram function within the “rms” package was used to build a regression model for key genes associated with MDD.

### 2.5. Immune‐Cell Infiltration Analysis

To calculate the score of infiltrating immune cells, the “CIBERSORT” algorithm was employed to transform the normalized gene expression matrix into the composition of infiltrating immune cells. The CIBERSORT deconvolution algorithm calculates this based on the ratio of RNA matrix to immune cells in the body. The Wilcoxon assay was utilized to compare the relative content of various immune cells in MDD group tissues and normal tissue samples under different modification modes. Additionally, the relationship between key genes and immune‐cell infiltration was investigated through Spearman correlation analysis. The proportion of immune cells in the MDD group and control group was visualized using the “vioplot” R package [18].

### 2.6. Single‐Nucleus Analysis

snRNA‐seq data were obtained from the GEO database (GSE144136). The Series Matrix file containing manually annotated nuclei was downloaded and analyzed using the Seurat package [18]. The Series Matrix file containing manually annotated nuclei was analyzed using the Seurat package. Standard quality‐control procedures were applied to remove nuclei with extremely low gene counts or abnormally high transcript counts, thereby reducing the influence of low‐quality nuclei and potential doublets. Gene expression matrices were log‐normalized following library size correction, and highly variable genes were identified for subsequent dimensionality reduction and clustering analyses. Data integration across samples was performed using Seurat′s standard integration workflow to minimize technical variability.

Principal component analysis (PCA) was conducted on the scaled data, and the top principal components were used to construct a shared nearest‐neighbor graph. Nuclei were clustered using the FindNeighbors and FindClusters functions with an empirically determined resolution parameter that balanced cluster granularity and biological interpretability. Cell‐type annotation was performed based on established canonical marker genes for major brain cell populations, including excitatory neurons, inhibitory (Inhibit) neurons, astrocytes (Astros), oligodendrocytes (Oligos), microglia (Micro), and endothelial cells. The VlnPlot function was used to visualize the expression patterns of representative marker genes and candidate biomarkers across identified cell types, and the RunTSNE function was applied for two‐dimensional visualization. Cell type–specific expression patterns were subsequently examined in the context of MDD versus control status.

### 2.7. Animal

Sprague–Dawley rats (Vital River Laboratories, Beijing, China, aged 6–8 weeks, body weight of 200 ± 250 g) were accommodated at the Laboratory Animal Center affiliated with the Hunan University of Chinese Medicine (HNUCM), ensuring continuous access to feed and water. All operations were in accordance with the management and ethics regulations of the Institutional Animal Care and Use Committee of HNUCM on the use of experimental animals (HNUCM21‐2404‐19).

### 2.8. Establishment of MDD Models

Twelve rats were randomly divided into normal control group (K) and CUMS treatment group (M). The rats of each cage were tilted for 24 h, swimming in cold water at 6°C for 5 min, water prohibition or fasting for 24 h, horizontal shaking for 15 min, tail clamping for 1 min, heat stress at 45°C for 5 min, alternating light/dark for 24 h, and the same stressor was given discontinuously for 6 weeks.

### 2.9. Sucrose Preference Test (SPT)

SPT takes place on Day 43. The experimental rats were involved in a 12‐h fast prior to the SPT, then followed by a 24‐h feeding period with two standard drinking bottles containing 1% sucrose solution and tap water. To prevent positional adaptation, the bottles were rotated every 6 h. The intake of sucrose and water was recorded at the end of the SPT, and the sucrose preference rate was presented as the percentage of sucrose intake/sucrose intake plus water intake.

### 2.10. Open‐Field Test (OFT)

The OFT was carried out on Day 43. In an 80 × 80 × 40 − cm open‐box testing platform, the bottom surface was divided into 16 equal‐sized square zones, and the rats were quickly positioned at the center of the box following device assembly. Movement trajectory tracking, total distance traveled, and the number of times each rat passed through the central grid were recorded for each experimental session. After 5 min of observation, the video recording was terminated, and the inner walls and bottom of the box were systematically disinfected using 75% ethanol. The testing platform was then thoroughly dried to eliminate any residual urine or odor that might interfere with subsequent measurements.

### 2.11. Real‐Time Quantitative PCR (qRT‐PCR)

Total RNA was isolated by Trizol method. cDNA was synthesized by PrimeScript RT Reagent Kit (Takara, Japan). qPCR reaction was performed using TB Green Premix Ex Taq (Takara, Japan). The following specific primers were used: DACH1 (forward: 5‐CCTGGGAAACCCGTGTACTC‐3, reverse: 5‐AGATCCACCATTTTGCACTCATT); FZD7 (forward: 5‐GCCACACGAACCAAGAGGAC‐3, reverse: 5‐CGGGTGCGTACATAGAGCATAA‐3); GULP1 (forward: 5‐CTTGGCAGTACCGAAGTGGAG‐3, reverse: 5‐TCCTTTGTTTTGGGTTCGAGAA‐3). GAPDH (forward: 5‐AGGTCGGTGTGAACGGATTTG‐3, reverse: 5‐GGGGTCGTTGATGGCAACA‐3). The GAPDH gene was used as an internal control to normalize gene expression. The 2−△△Ct method was used to calculate the relative expression levels.

### 2.12. Western Blot

All proteins extracted from the brain tissues of each group were lysed using RIPA buffer. The supernatant was obtained by high‐speed centrifugation at 4°C, and the protein level was analyzed using the Bicinchoninic Acid Assay Kit. Next, the proteins were separated one by one through electrophoresis and transferred onto the PDVF membrane. Then it is sealed with a special sealing solution. Next, the obtained membrane will be paired with the corresponding primary antibodies, including DACH1 (1:5000), GULP1 (1:1000), and FZD7 (1:1000) and refrigerated overnight. The conjugated antibodies were detected with appropriate secondary antibodies, and chemiluminescence imaging was used. The protein bands were processed using ImageJ software.

### 2.13. Statistical Analysis

All data were presented as the mean ± standard deviation and analyzed using the R programming language and SPSS Version 26.0 software. Comparisons between groups and within groups were conducted using the Wilcoxon test. The results from body weight, FST, and OFT were analyzed by unpaired (independent) Student′s *t*‐test for two‐group comparisons. Unless otherwise specified, a *p* value less than 0.05 was considered statistically significant.

## 3. Results

### 3.1. Target Module and Gene Screening Via WGCNA

In the retrospective analysis, GSE53987 and GSE92538 were amalgamated, encompassing 79 MDD patients and 111 control subjects. The integrated dataset unveiled 132 DEGs, comprising 118 downregulated and 14 upregulated genes (Figure 2a,b). Employing a soft threshold (*β* = 17) that exhibited optimal scale‐free fitting metrics and average connectivity, a standard scale‐free network was constructed (Figure 2c). Utilizing average connected hierarchical clustering with a minimum module size of 50, genes were grouped based on the mixed dynamic pruning tree criterion (Figure 2d). Eight modules were successfully identified (Figure 2e). The cyan (*r* = −0.34, *p* = 2*e* − 06), green (*r* = −0.22, *p* = 0.002), and gray (*r* = 0.27, *p* = 1*e* − 04) modules displayed the highest correlation with MDD. The intersection of the first two modules with DEGs yielded a total of 25 genes.

Figure 2(a) Heatmap plot of differentially expressed genes for conditions of interest. (b) Volcano plot of differentially expressed genes for conditions of interest. (c) Analysis of scale‐free fit indices and average connectivity for various soft threshold values of power. (d) The gene hierarchical clustering dendrogram. (e) Relationships of consensus modules with diseases. Each specified color represents a specific gene module. (f) GO term analysis of DEGs. (g) KEGG term analysis of DEGs.

**Figure:**
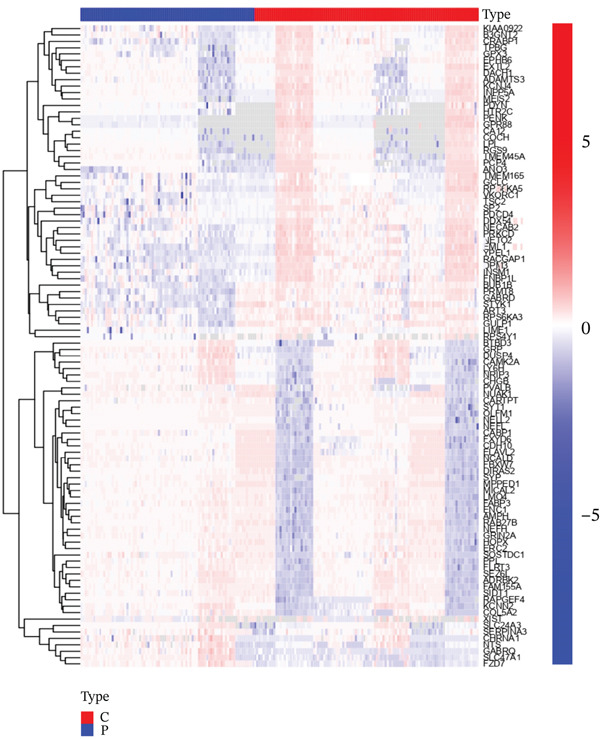
(a)

**Figure:**
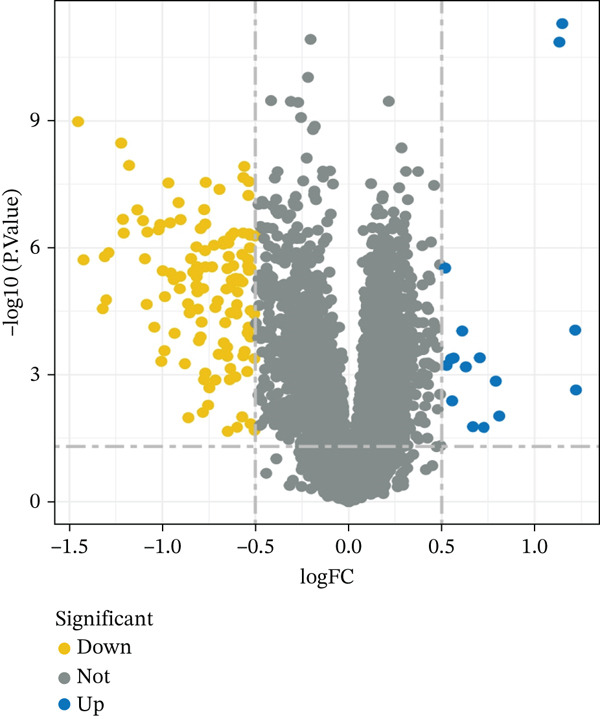
(b)

**Figure:**
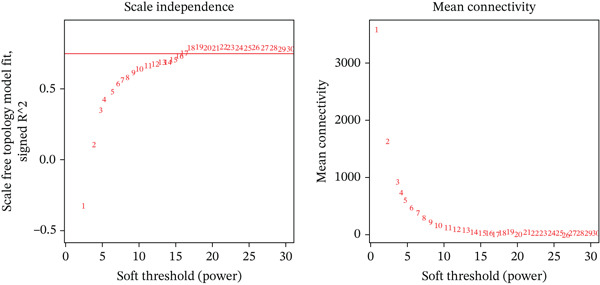
(c)

**Figure:**
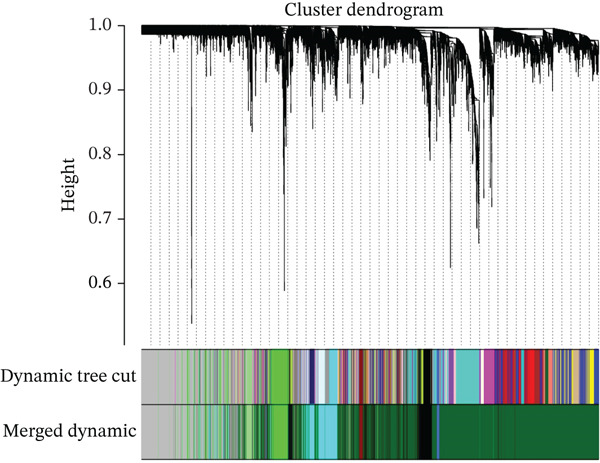
(d)

**Figure:**
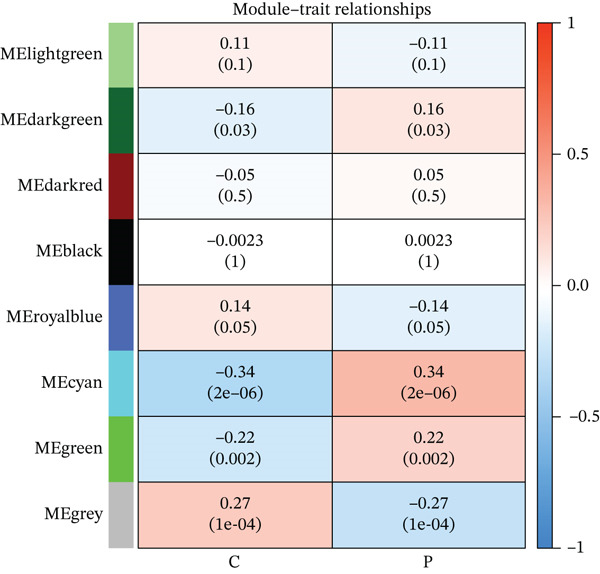
(e)

**Figure:**
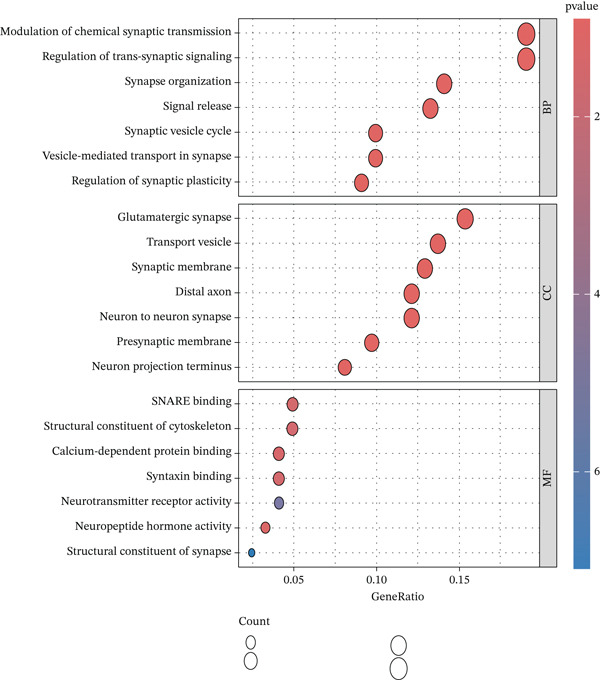
(f)

**Figure:**
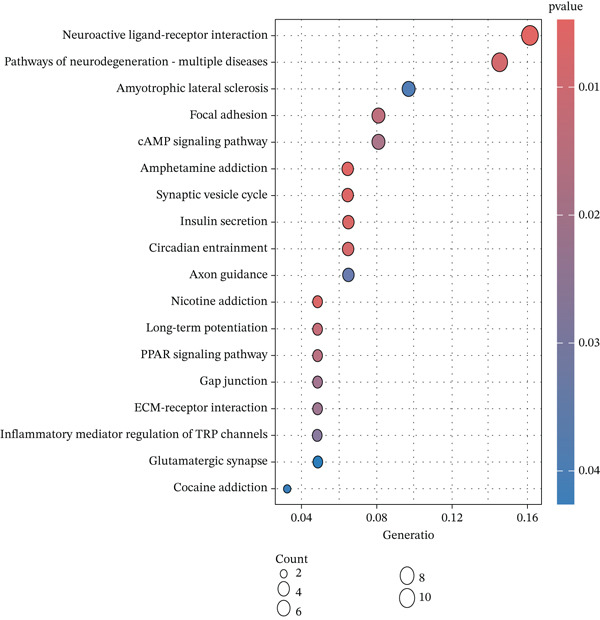
(g)

### 3.2. Functional Enrichment

The GO enrichment analysis revealed significant enrichment of the 25 genes in various biological processes, including signal release, synapse organization, synaptic vesicle cycle, and regulation of trans‐synaptic signaling (Figure 2f).

The KEGG enrichment analysis demonstrated that these 25 genes were enriched in relevant pathways such as neuroactive ligand–receptor interaction, cAMP signaling pathway, suggesting potential involvement in neuroimmune‐related signaling pathways (Figure 2g). Additionally, pathways like circadian entrainment, insulin secretion, nicotine addiction, and synaptic vesicle cycle were also identified.

### 3.3. Identification of Diagnostic Markers in MDD

Through the LASSO regression algorithm, eight genes were identified as potential biomarkers (Figure 3a). SVM‐RFE algorithm achieved the highest accuracy with 23 feature genes, reaching 0.768 (Figure 3b). The RF algorithm identified 10 candidate genes (Figure 3c). Ultimately, three genes (DACH1, FZD7, and GULP1) emerged as diagnostic markers for MDD (Figure 3d). In comparison with the healthy control group, DACH1 and GULP1 exhibited significant upregulation in MDD patients, whereas FZD7 was markedly downregulated (Figure 4a,b).

Figure 3(a) The LASSO logistic regression algorithm was used to screen hub genes. (b) The SVM‐RFE algorithm was used to screen hub genes. (c) Unveiling hub genes through RF algorithms. (d) Venn diagram showed the intersection of diagnostic markers obtained by the three algorithms.

**Figure:**
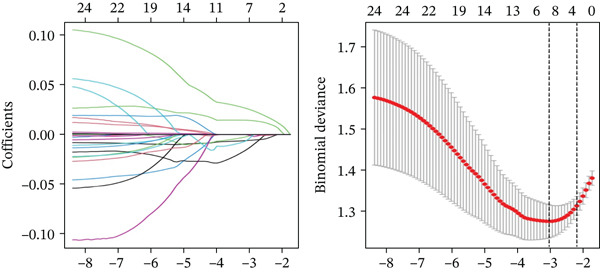
(a)

**Figure:**
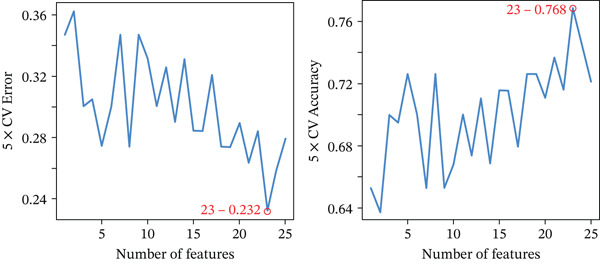
(b)

**Figure:**
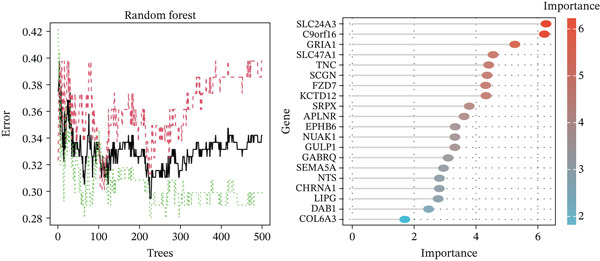
(c)

**Figure:**
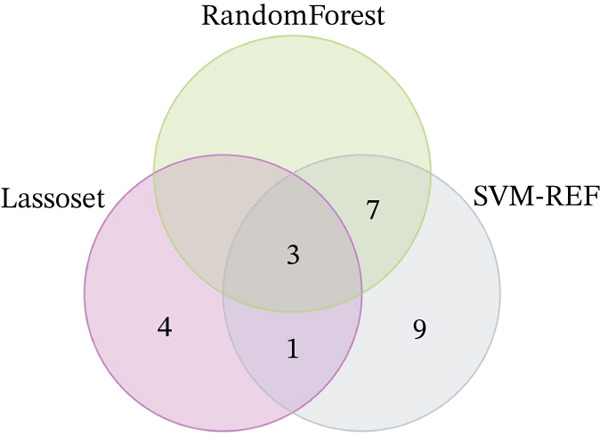
(d)

Figure 4(a, b) The expressing pattern of three genes in MDD and control groups,  ^∗∗∗^ *p* < 0.001,  ^∗∗^ *p* < 0.01, and  ^∗^ *p* < 0.05.

**Figure:**
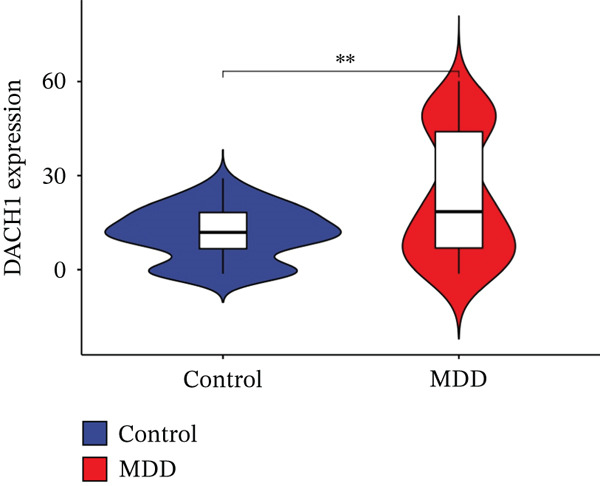
(a)

**Figure:**
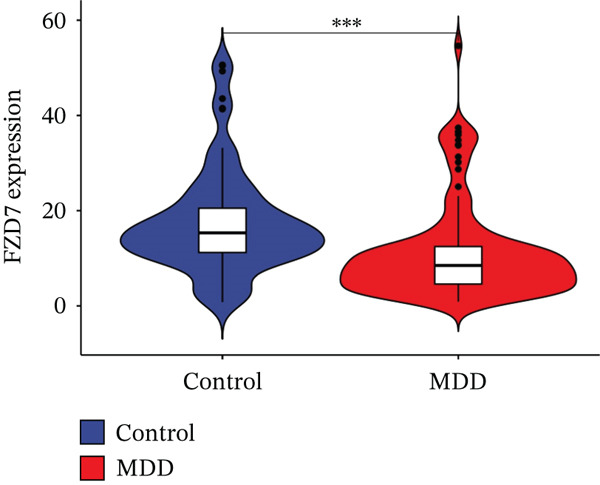
(b)

**Figure:**
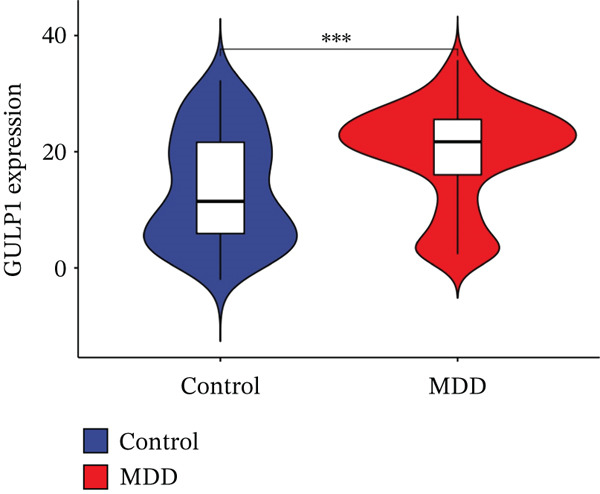
(c)

**Figure:**
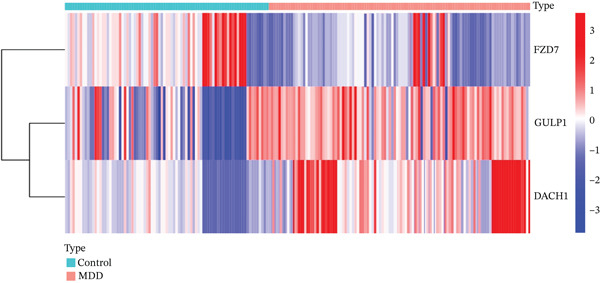
(d)

### 3.4. Nomogram Construction and ROC Evaluation

Constructing a nomogram to assess the significance of DACH1, FZD7, and GULP1 in diagnosing MDD (Figure 5a), the calibration curve indicates that the nomogram exhibits high predictive accuracy, with minimal deviation between the actual risk and predicted risk of MDD (Figure 5b).

Figure 5(a) A nomogram for MDD based on the three genes. (b) Construction of a calibration curve. (c–h) ROC assays for three genes based on validation dataset.

**Figure:**
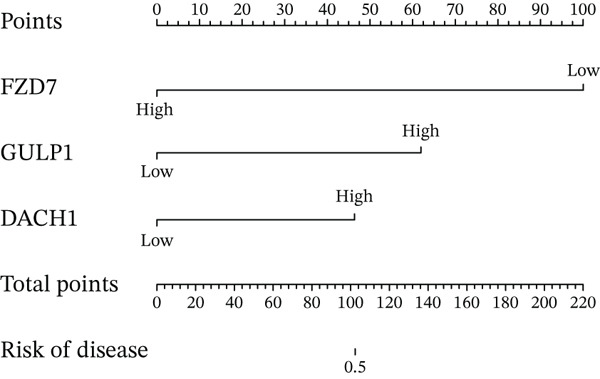
(a)

**Figure:**
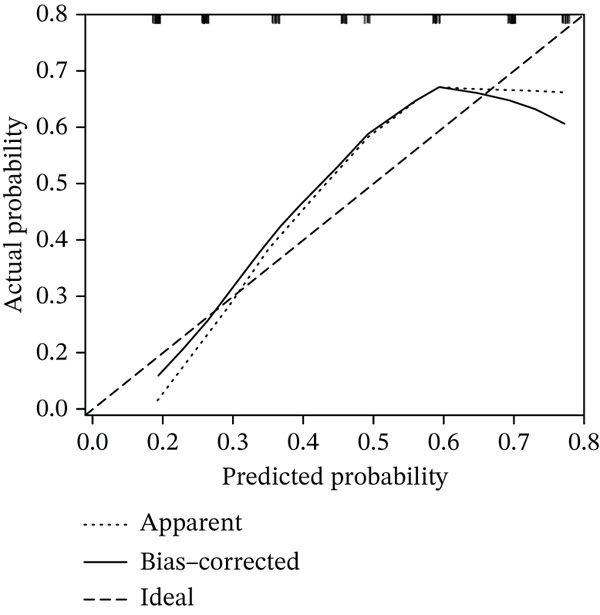
(b)

**Figure:**
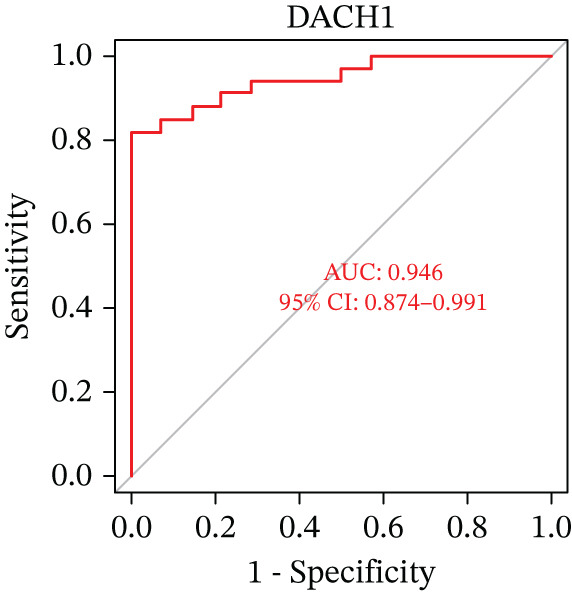
(c)

**Figure:**
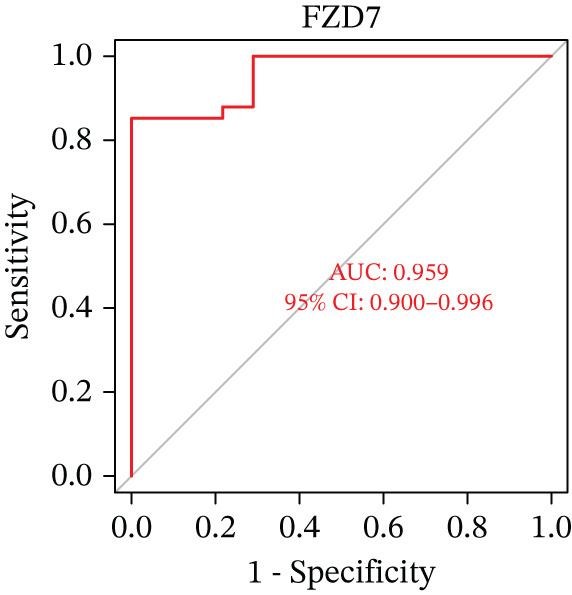
(d)

**Figure:**
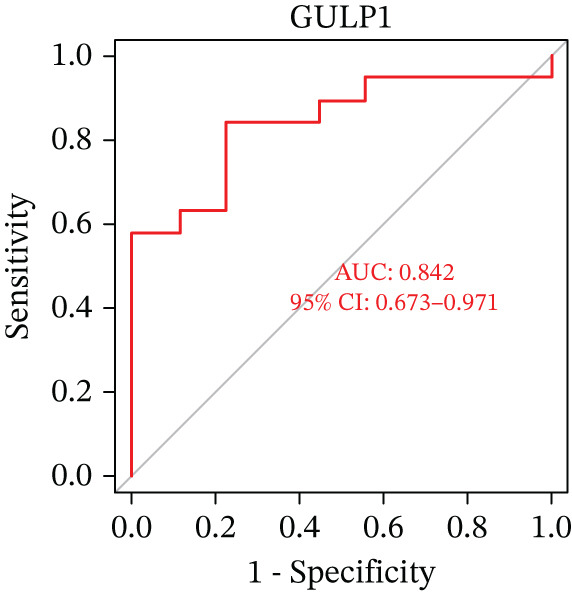
(e)

**Figure:**
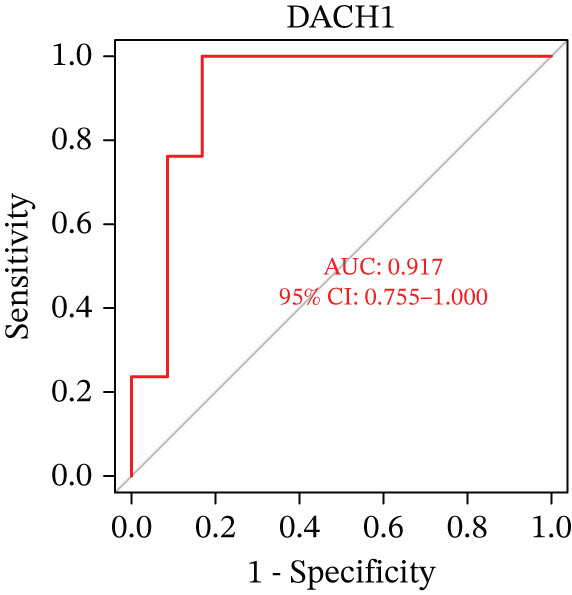
(f)

**Figure:**
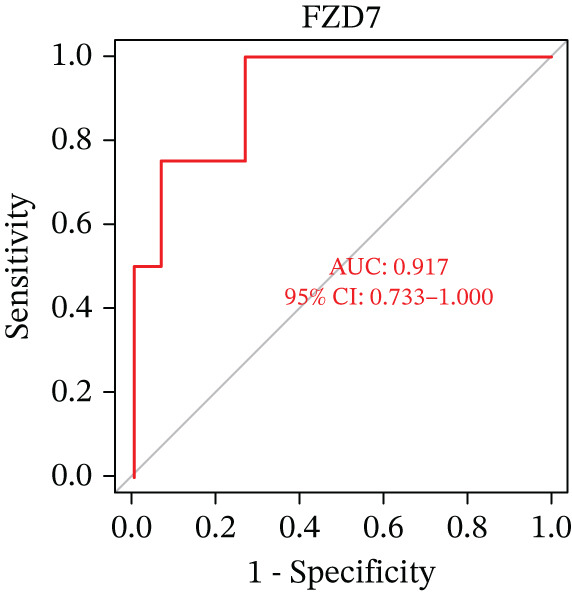
(g)

**Figure:**
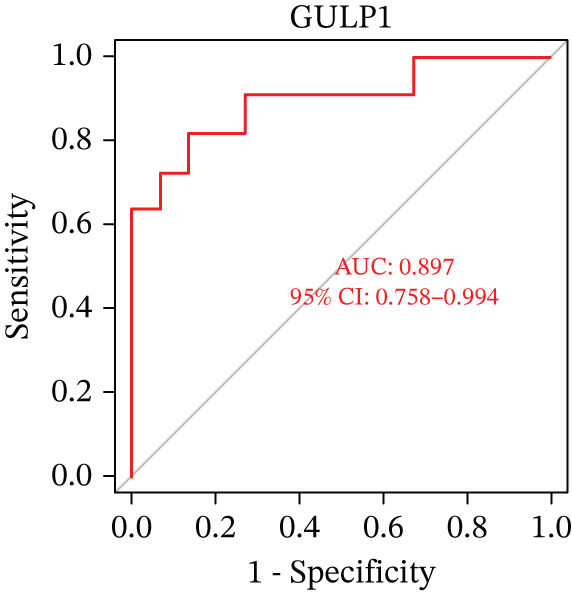
(h)

Evaluation of the diagnostic value of each candidate hub gene was performed using ROC curves. The AUC and 95% CI for each gene are presented in Figures 5c, 5d, and 5e: DACH1 (AUC: 0.946, 95% CI: 0.874–0.991), FZD7 (AUC: 0.959, 95% CI: 0.900–0.996), and GULP1 (AUC: 0.842, 95% CI: 0.673–0.971). To validate the findings, GSE54568 was employed as the validation set, and Figures 5f, 5g, and 5h demonstrates the AUC and 95% CI: DACH1 (AUC: 0.917, 95% CI: 0.755–1.000), FZD7 (AUC: 0.917, 95% CI: 0.733–1.000), and GULP1 (AUC: 0.897, 95% CI: 0.758–0.994). The ROC curve analysis reveals that all genes exhibit satisfactory diagnostic performance.

In addition to ROC analysis, DCA was conducted to assess the potential clinical utility of these genes as diagnostic markers. The DCA curves (Figure S1) indicate that incorporating these genes into a diagnostic model provides a net clinical benefit across a range of threshold probabilities, further supporting their translational value. Taken together, these analyses suggest that DACH1, FZD7, and GULP1 are promising diagnostic biomarkers for MDD.

### 3.5. Immune Infiltration Analysis

To delve deeper into the intricate relationship between biomarkers and levels of immune infiltration landscape in MDD patients, an immune‐cell correlation analysis was performed comparing the MDD group with the healthy control group (Figures 6a, 6b, and 6c). The findings underscored a substantial correlation between the MDD group and the healthy control group.

Figure 6(a) Bar plot shows the fraction of the 22 types of immune cells in the control and MDD groups. (b) Correlation analysis of the 22 types of immune cells. (c) Violin diagram shows the fraction of 22 types of immune cells in the control and MDD groups. (d–f) Correlation graphs of 22 immune cells with DACH1, FZD7 and GULP1.

**Figure:**
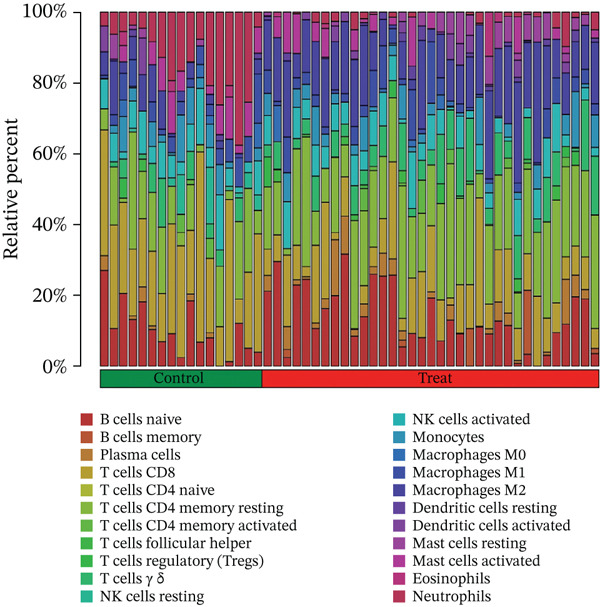
(a)

**Figure:**
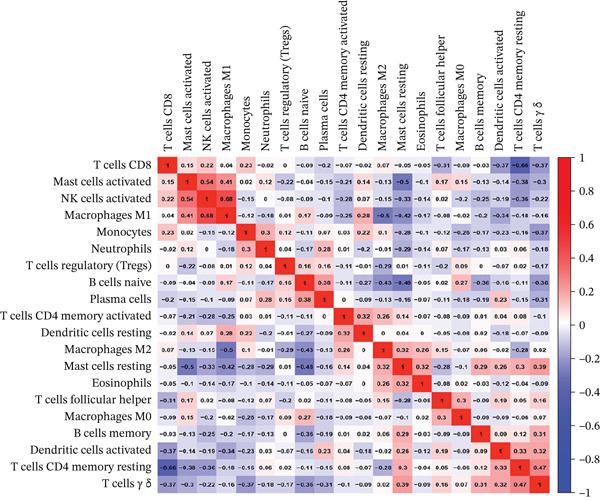
(b)

**Figure:**
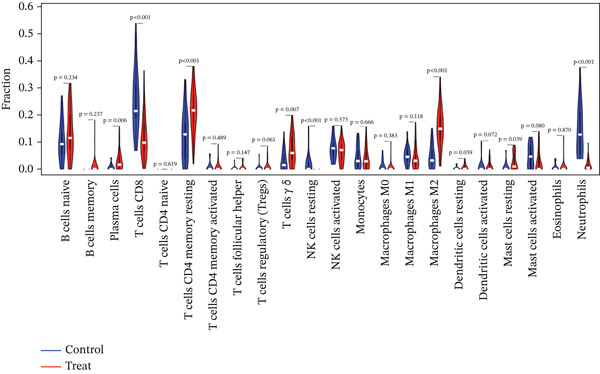
(c)

**Figure:**
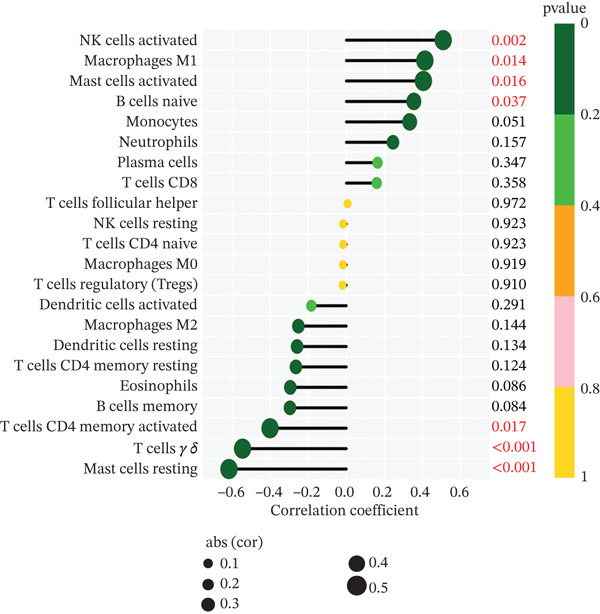
(d)

**Figure:**
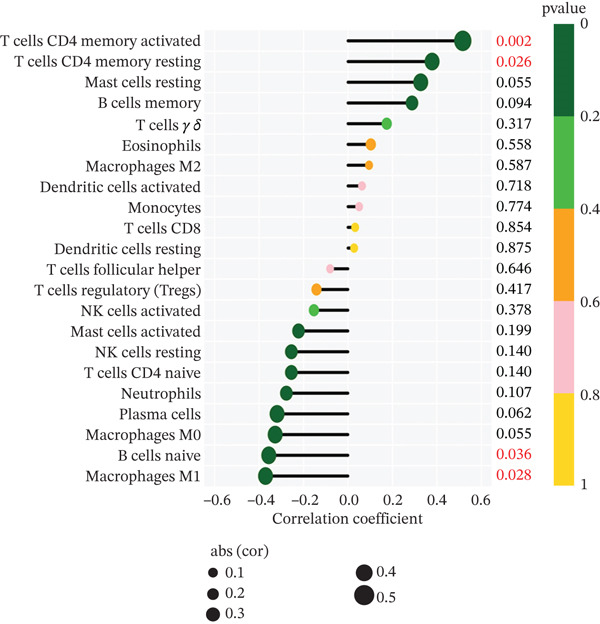
(e)

**Figure:**
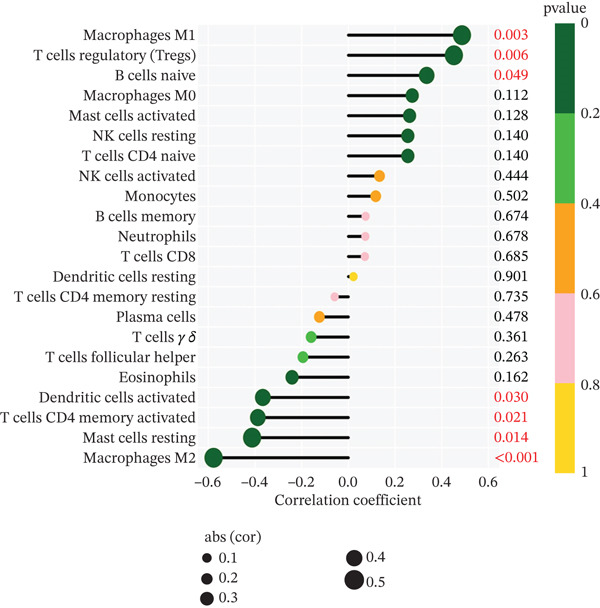
(f)

Subsequently, the investigation extended to exploring the interplay between three distinct biomarkers (DACH1, FZD7, and GULP1) and levels of immune cells (Figures 6d, 6e, and 6f). Specifically, DACH1 exhibited a positive correlation with naïve B cells (*R* = 0.35, *p* = 0.037), activated mast cells, M1 macrophages (*R* = 0.41, *p* = 0.014), and activated NK cells (*R* = 0.51, *p* = 0.0021). Conversely, it showed a negative correlation with resting mast cells (*R* = −0.62, *p* = 0.000082), gamma delta T cells (*R* = −0.54, *p* = 0.00072), and activated CD4 memory T cells (*R* = −0.4, *p* = 0.017) (Figure 7a). FZD7 demonstrated a positive correlation with resting CD4 memory T cells (*R* = 0.38, *p* = 0.026) and activated CD4 memory T cells, while displaying a negative correlation with M1 macrophages (*R* = −0.37, *p* = 0.028) and naïve B cells (*R* = −0.36, *p* = 0.036) (Figure 7b). GULP1 exhibited a positive correlation with naïve B cells (*R* = 0.34, *p* = 0.049), regulatory T cells (*R* = 0.45, *p* = 0.0064), and resting mast cells (*R* = 0.4, *p* = 0.016), and concurrently, a negative correlation with M2 macrophages (*R* = −0.58, *p* = 0.00036), activated CD4 memory T cells, and activated dendritic cells (*R* = −0.37, *p* = 0.03) (Figure 7c).

Figure 7Results of correlation analysis between biomarkers ((a) DACH1, (b) FZD7, and (c) GULP1) and immune‐cell infiltration in MDD patients. Correlation coefficients *R* > 0 indicated positive correlation, *R* < 0 indicated negative correlation, and *p* < 0.05 indicated significant difference.

**Figure:**
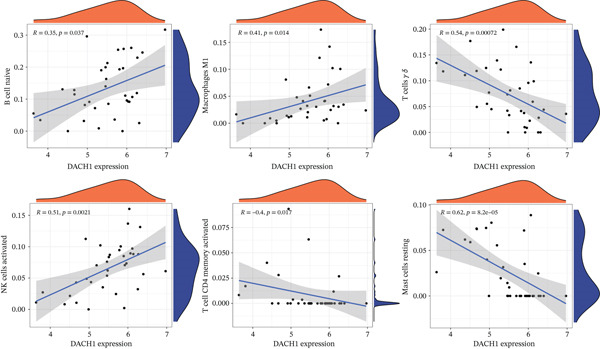
(a)

**Figure:**
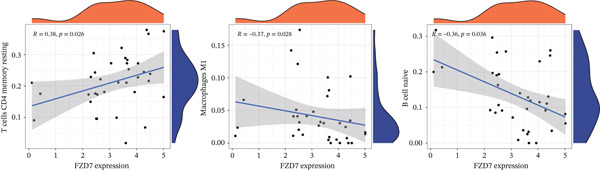
(b)

**Figure:**
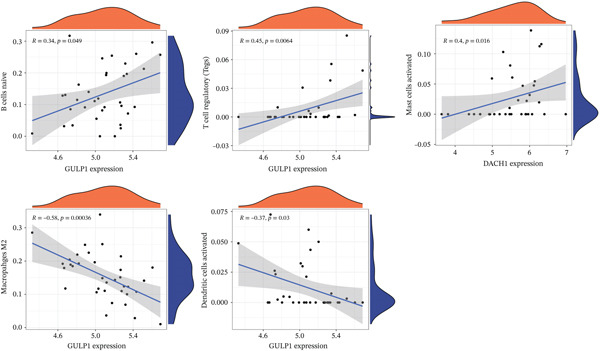
(c)

### 3.6. Single‐Nucleus Data Analysis

To explore alterations in target genes within specific cell populations of MDD patients, an analysis of snRNA‐seq data from brain tissue biopsy samples of both healthy individuals and MDD patients was conducted. The annotated results encompassed eight cell types: Astros, endothelium (Endo), Oligos, oligodendrocyte precursor cell (OPC), Micro, excessory (EX) neuron, Inhibit neuron, and mixed cells combining various cell types (Figure 8a).

Figure 8(a) The MDD and control samples were categorized into eight cell types: astrocyte (Astros), endothelium (Endo), oligodendrocyte (Oligos), oligodendrocyte precursor cell (OPC), microglia (Micro), excitatory neurons (EX), inhibitory (Inhibit) neurons, and mixed cell (Mix). (b–d) Expression distribution maps for DACH1, FZD7, and GULP1.

**Figure:**
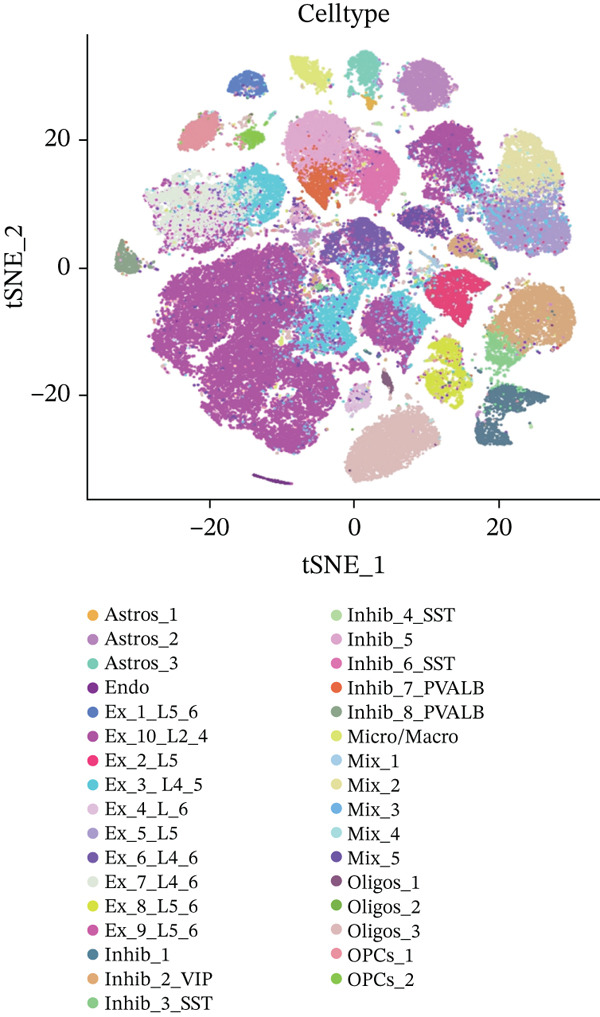
(a)

**Figure:**
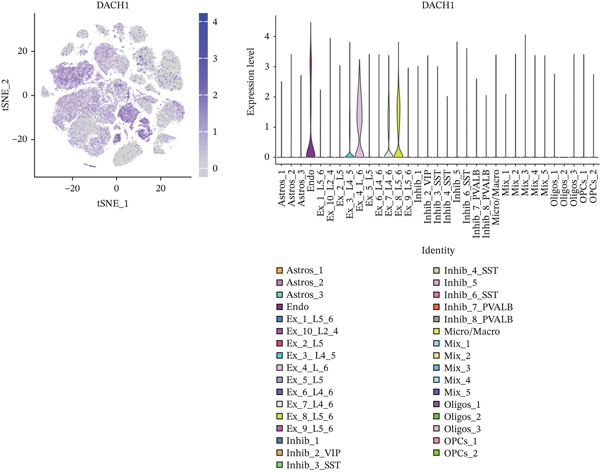
(b)

**Figure:**
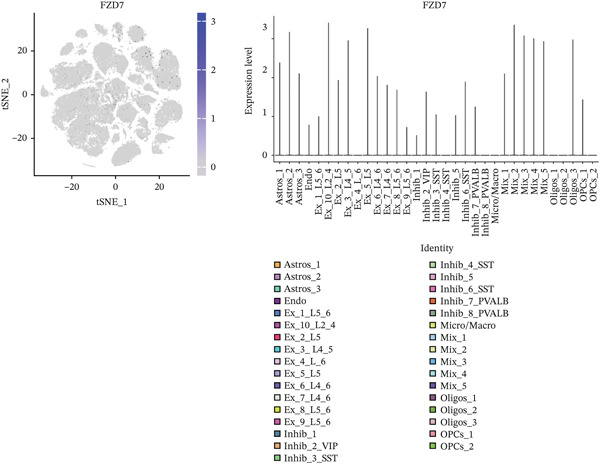
(c)

**Figure:**
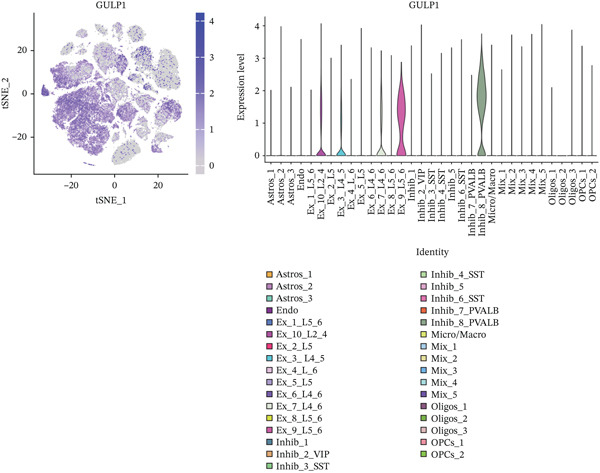
(d)

Visualization using TSNE revealed distinct expression patterns. Specifically, DACH1 exhibited predominant expression in endothelial cells and epithelial neuron cell types (Figure 8b). FZD7 showed enrichment in Astros cell types (Figure 8c). Meanwhile, GULP1 demonstrated higher expression levels in excitatory neurons and Inhibit neurons cell types (Figure 8d). Studies with the larger sample size are needed for verification in the future.

### 3.7. Validation of the MDD Model

As shown in Figure 9a, compared with the control group, the CUMS group showed a significant reduction in body weight at the beginning of the experiment, with the lowest body weight at Week 6 (*p* < 0.001). The reduced preference for sucrose in rats reflects the development of anhedonia, one of the key symptoms in patients with MDD. CUMS treatment for 6 weeks significantly reduced the sucrose preference index of SPT in rats (*p* < 0.001) in Figure 9b. OFT responded to the behavioral responses of the rats. The total distance (*p* < 0.05), number of times crossed the central grid (*p* < 0.05), and cumulative times of center‐point (*p* < 0.05) of OFT were significantly reduced in CUMS‐treated rats compared with the control group (Figures 9c, 9d, 9e, and 9f). Together, these data indicate that CUMS induces MDD‐like behaviors.

Figure 9(a) Body weight of the control and CUMS‐treated rats. (b) The sucrose preference index of SPT. (c) Representative running trace in OFT, the observation time was 10 min. (d–f) (d) Total running distance, (e) number of crossings of the central grid, and (f) center‐point cumulative duration of OFT. All data are presented as mean ± standard deviation (*n* = 6).  ^∗^ *p* < 0.05,  ^∗∗^ *p* < 0.01,  ^∗∗∗^ *p* < 0.001, compared with the control group (K group).

**Figure:**
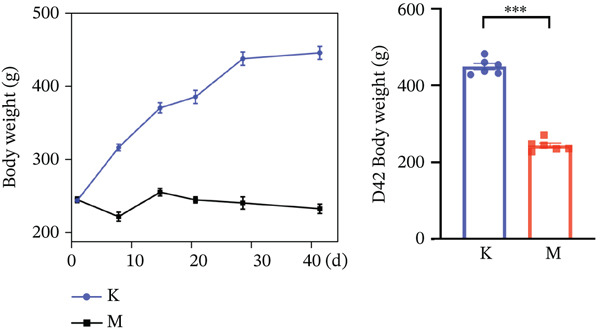
(a)

**Figure:**
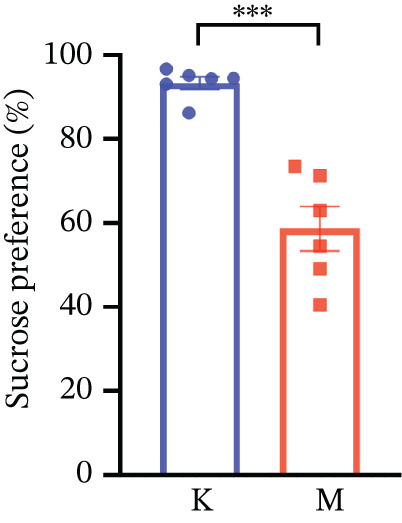
(b)

**Figure:**
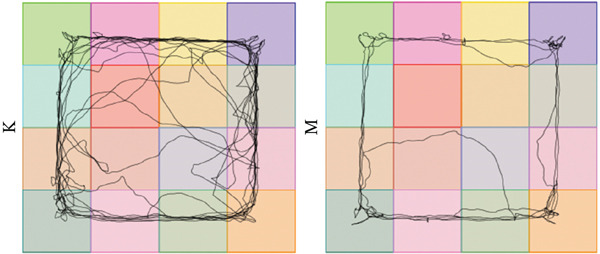
(c)

**Figure:**
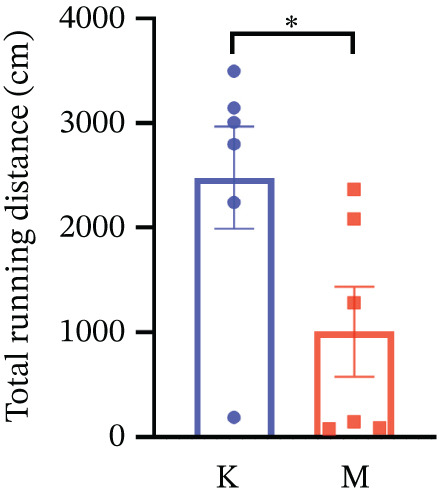
(d)

**Figure:**
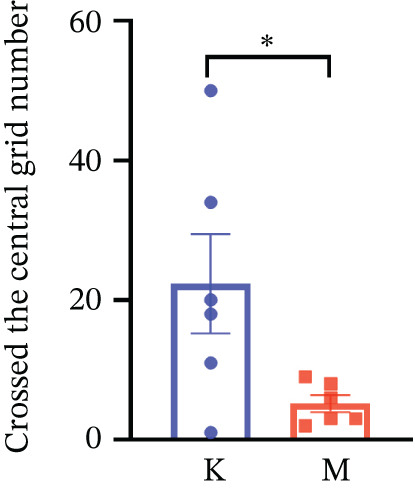
(e)

**Figure:**
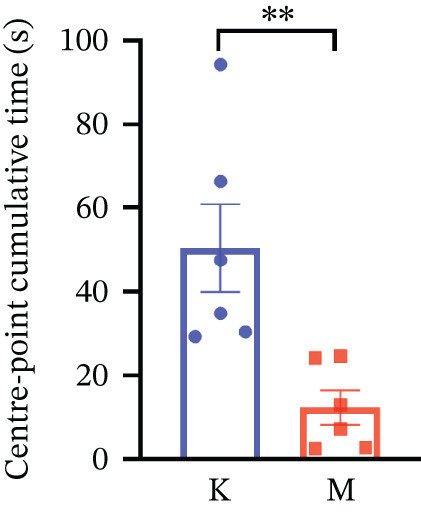
(f)

### 3.8. Transcriptome Analysis and Validation Results

To further clarify the molecular mechanisms regulating MDD, the brain tissues from control and CUMS rats were collected and RNA was isolated for transcriptome analysis. The RNA expression profiles of the two groups were significantly different, as determined by PCA (Figure 10a). There were 470 DEGs between the control and CUMS groups, of which 205 DEGs were upregulated and 265 DEGs were downregulated (Figure 10b). Based on this difference, GO analysis was performed to determine the biological function of the proteins encoded by DEGs (Figure 10c). DEGs between the two groups exhibited enrichment in biological processes, including regulation of ERK1 and ERK2 cascades (GO:0070372), histamine secretion involved in inflammatory responses (GO:0002441), and synaptic pruning (GO:0098883). DEGs between the two groups exhibited enrichment in cellular components, including outer plasma membrane (GO:0009897), outer coat structure (GO:0030312), receptor complex (GO:0043235). DEGs between the two groups exhibited enrichment in molecular functions, including receptor–ligand activity (GO:0048018), signaling receptor–activator activity (GO:0030546), and enzyme–inhibitor activity (GO:0004857). KEGG enrichment analysis further identified pathways associated with DEG, including cell adhesion molecules, neuroactive ligand–receptor interactions, antigen processing and presentation, IL‐17 signaling pathway, leukocyte migration across the Endo, HIF‐1 signaling pathway, relaxin signaling pathway, and intestinal immune network (Figure 10d). This further suggests that MDD is significantly associated with the immune system. As shown in the results of Figure 10b, DACH1 and GULP1 were upregulated, whereas FZD7 was downregulated in the brain tissues of CUMS‐induced depression model rats.

Figure 10(a) PCA showing the DEGs between the control and CUMS group. (b) Volcano plot showing genes that were downregulated or upregulated in the CUMS group and the criteria were set at logFC > 1 and FDR *p* < 0.05. (c) Heatmap visualization of the DEGs between the control and CUMS group within pathways identified by GO enrichment analysis. (d) Heatmap visualization of the DEGs between the control and CUMS group within pathways identified by KEGG enrichment analysis. (e) qRT‐PCR analysis of three key differentially expressed genes. (f) Western blot analysis of three key differentially expressed genes. All data are presented as mean ± standard deviation (*n* = 3).  ^∗∗^ *p* < 0.01, compared with the control group (K group) ^∗∗^.

**Figure:**
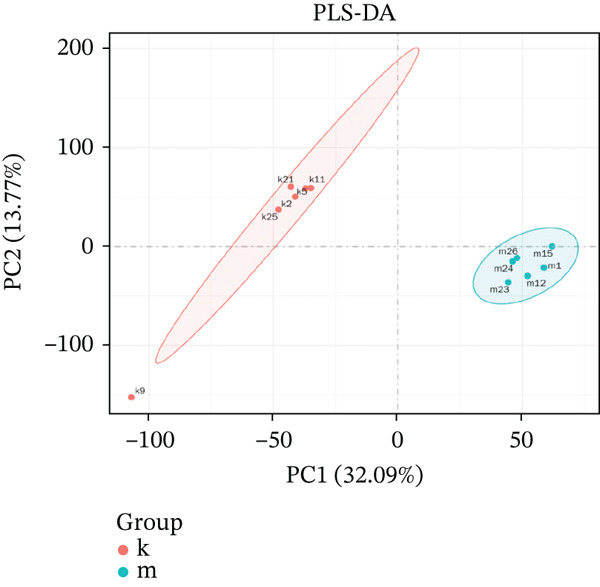
(a)

**Figure:**
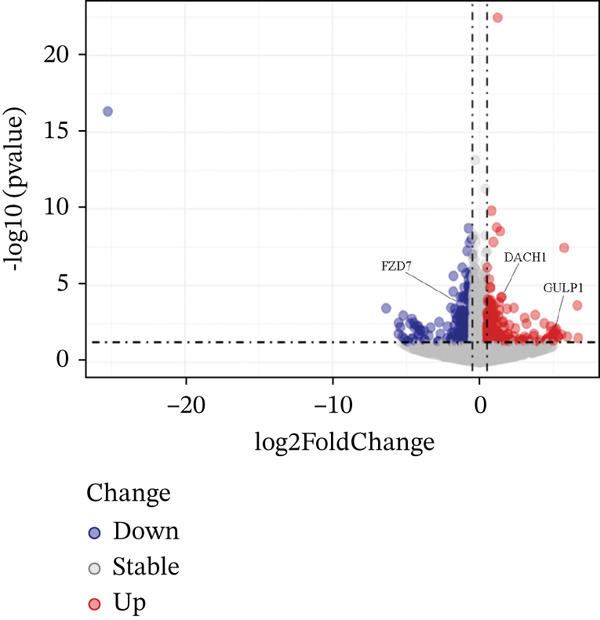
(b)

**Figure:**
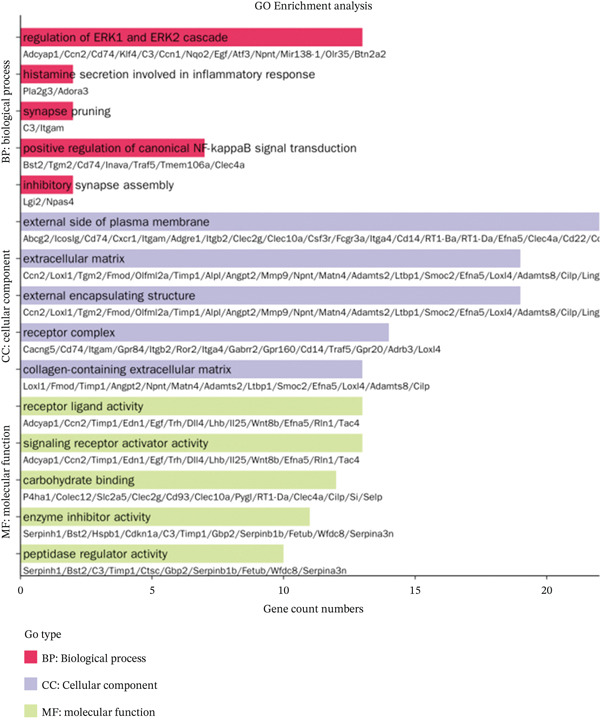
(c)

**Figure:**
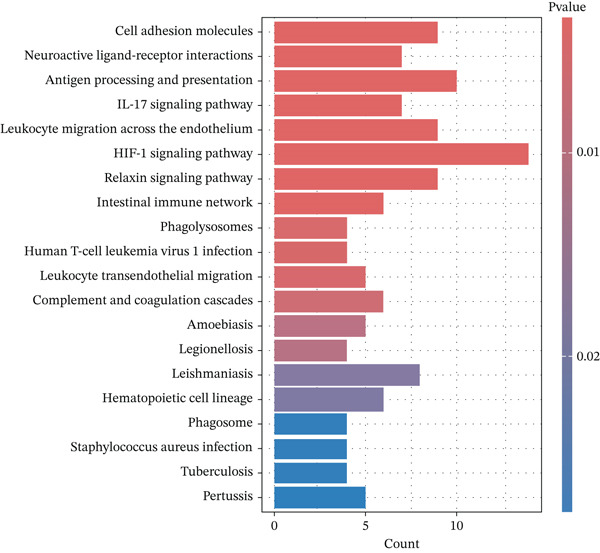
(d)

**Figure:**
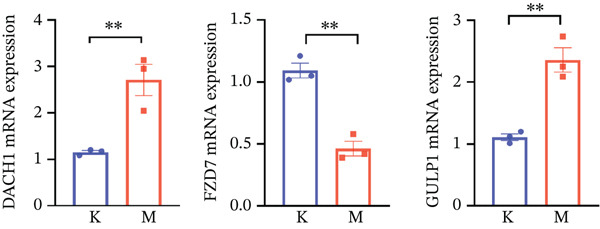
(e)

**Figure:**
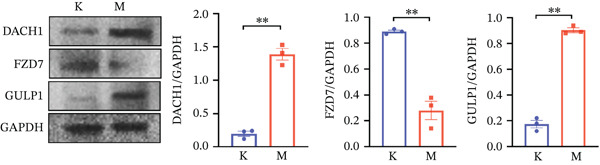
(f)

We therefore collected brain tissues from control rats and CUMS‐induced depression model rats for qRT‐PCR and western blot validation. As shown in Figure 10e,f, compared with the brain tissues of control rats, the mRNA and protein expression of DACH1 and GULP1 were highly expressed, whereas the mRNA and protein expression of FZD7 was lowly expressed in the brain tissues of CUMS‐induced depression model rats. This quantitative analysis result is consistent with the database analysis results and transcriptome sequencing results mentioned above, indicating that our database analysis results and transcriptome sequencing data are accurate and reliable, and can be used as reference data for future studies. It can be concluded that the key DEGs (DACH1, FZD7, and GULP1) detected in this study are important for studying the molecular mechanism of MDD.

## 4. Discussion

Given the escalating pressures of work and life, the annual rise in MDD prevalence imposes not only suffering on patients but also varying burdens on their friends and family. The pathophysiology and etiology of MDD are exceedingly intricate. Over the past few decades, mounting evidence has indicated a correlation between immune imbalance and severe depression. Clinical research findings establish a link between the pathophysiology of MDD and the immune system [9, 19]. Both peripheral and central immune functions undergo alterations in MDD patients [20], underscoring the potential significance of the immune system pathogenesis in MDD development.

To delve deeper into the relationship between biomarkers and the immune infiltration landscape in MDD patients, we employed the CIBERSORT method for a comprehensive assessment of immune‐cell infiltration roles. Examining immune infiltration patterns in both MDD and normal tissues revealed distinct associations.

DACH1 exhibited a positive correlation with naïve B cells, activated mast cells, M1 macrophages, and activated NK cells. Conversely, it showed a negative correlation with resting mast cells, gamma delta T cells, and activated CD4 memory T cells. FZD7 demonstrated a positive correlation with resting CD4 memory T cells and activated CD4 memory T cells, while displaying a negative correlation with M1 macrophages and naïve B cells. GULP1 exhibited a positive correlation with naïve B cells, regulatory T cells, and M1 macrophages, along with a negative correlation with M2 macrophages, resting mast cells, activated CD4 memory T cells, and activated dendritic cells. To further validate the relationship between biomarkers and MDD immune infiltration, we constructed a CUMS‐induced animal model. After the successful establishment of this model, RNA from brain tissues was extracted for transcriptomic sequencing, and it was found that DACH1 and GULP1 were upregulated in brain tissues of CUMS‐induced depression model rats, whereas FZD7 was downregulated. Analysis of the sequencing results indicated a trend of association between MDD and immune‐cell infiltration, which requires further investigation to clarify causal mechanisms. In addition, the qRT‐PCR and western blot results were consistent with the above database analysis and transcriptome sequencing results.

DACH1, a pivotal member of the Ski gene family, is widely distributed across various human tissues, with a prominent presence in vital organs like the brain and heart. Its primary role involves precise control of gene expression, achieved through direct binding to target genes or interaction with other regulatory factors [21]. Studies have previously highlighted the substantial expression of DACH1 in proliferative neurons within the cortical, subventricular, and striatal regions of the brain [22]. Furthermore, DACH1 plays a role in endothelial cells and the nerve plexus, aligning with our observations in monocyte studies [23]. Additional research indicates its potential as a novel therapeutic approach for chronic obstructive pulmonary disease treatment [24]. Previous studies have reported an association between DACH1 expression and activated CD4 T cells [25], which is consistent with the immune‐cell correlation patterns observed in our analysis. However, these findings should be interpreted cautiously, as they reflect associations rather than direct evidence of functional immune regulation. Although prior investigations have not delved into the interplay between DACH1 in MDD and endothelial and neural cells, it warrants further exploration to uncover its specific mechanisms.

FZD7, a member of the G protein–coupled receptor family, serves as a pivotal component in the 7‐transmembrane cell surface receptor and Wnt signaling pathway [26]. Playing a critical role in the regulation of stem cell pluripotency and cell differentiation, FZD7 integrates with other pathways and associated signaling molecules such as fibroblast growth factor and bone morphogenetic protein [27]. The Wnt signaling pathway, in which FZD7 is a key player, holds paramount importance in neural development and the modulation of the adult nervous system′s function and structure [28]. Fibroblast growth factors, along with bone morphogenetic proteins, are implicated in depression pathogenesis and contribute significantly to the maintenance of adult hippocampal neurogenesis, alongside brain‐derived neurotrophic factors, vascular endothelial growth factors, and other signaling pathways [29, 30]. Studies have indicated noteworthy differences in the gene expression of FZD7 in the proliferation of lymphoblast‐like cell lines in depression patients treated with depression drugs, suggesting FZD7′s potential as a gene expression biomarker for MDD [31]. Moreover, research has highlighted significant differences in mRNA expression of FZD7, a closely correlated gene in the Wnt signaling pathway, among MDD patients [32]. Notably, FZD7 expression has been identified in brain tissue endothelial cells, Astros, and neurons [33].

GULP1, functioning as a highly conserved and widely expressed adapter protein, intricately regulates intracellular signaling [34]. Its primary role involves active participation in cytoskeletal rearrangement, a pivotal process in embryonic development, tissue renewal, inflammation, and autoimmunity [35]. Furthermore, GULP1 plays a crucial role in the intracellular transport of ligands for lipoprotein receptor‐related Protein 1, thereby regulating cholesterol metabolism and engaging in the processing of amyloid precursor proteins and the production of amyloid proteins *β* [36]. In light of research indicating inflammation as a significant disease regulator contributing to susceptibility to depression [37], and recognizing the association between lipid disorders and the onset and progression of MDD in animal and clinical studies, we propose a speculative link between GULP1 and MDD. We speculate that GULP1 may be involved in biological processes related to inflammation and lipid metabolism that have been implicated in MDD. This hypothesis is based on indirect evidence and warrants further experimental validation.

However, several limitations of this study should be acknowledged. First, the analysis was based solely on datasets obtained from the GEO database, which may limit the sample size and generalizability of the findings. Second, the lack of clinical experimental validation restricts our ability to elucidate the precise biological mechanisms through which the immune‐related hub genes (DACH1, FZD7, and GULP1) may be involved in the progression of MDD. Therefore, future studies should incorporate larger, multidatabase cohorts and perform in‐depth mechanistic investigations to clarify the functional roles of these genes. In addition, immune cell infiltration estimated by CIBERSORT is derived from computational deconvolution of bulk transcriptomic data and represents relative immune‐cell proportions rather than direct measurements. Consequently, the observed immune‐cell correlations should be interpreted as associative rather than causal or indicative of specific immunological mechanisms.

## 5. Conclusion

Our study identified potential immune‐related hub genes (DACH1, FZD7, and GULP1) in MDD by leveraging the GEO database and transcriptomics. The integration of immune infiltration analysis and snRNA‐seq has broadened our comprehension of the link between MDD and immunity. Our findings identify prospective therapeutic targets and offer novel sides for the early clinical diagnosis and intervention of MDD. Nevertheless, for further validation of the feasibility of these biomarkers in MDD, future research endeavors should prioritize larger sample sizes and clinical trials.

## Author Contributions

**Long Kangsheng:** writing – original draft, visualization, software, resources, project administration, methodology, investigation, formal analysis, data curation, conceptualization. **Yang Xiaohui:** writing – original draft, visualization, software, resources, methodology, formal analysis, conceptualization. **Pei Xin:** writing – original draft, visualization, software, resources, methodology, formal analysis, conceptualization. **Ye Yong:** writing – original draft, validation, formal analysis, data curation. **Li Hongliang:** writing – review and editing, supervision, project administration, conceptualization. **Deng Yihui:** writing – review and editing, supervision, project administration, methodology, conceptualization, funding acquisition.

## Funding

This work was supported by grants from the Science and Technology Innovation Team Project of Hunan Province (2020RC4050), Hunan Provincial Health and Wellness Research Project (20257417), and Hunan Provincial Natural Science Foundation (2026JJ82467).

## Conflicts of Interest

The authors declare no conflicts of interest.

## Supporting information


**Supporting Information** Additional supporting information can be found online in the Supporting Information section. Figure S1: This figure illustrates the decision curve analysis (DCA) of the predictive model. Table S1: This table presents the relevant clinical data of patients with severe depression, which is used to analyze the differential genes and common pathways.

## Data Availability

The data that support the findings of this study are available on request from the corresponding author. The data are not publicly available due to privacy or ethical restrictions.
