# Supplementary material for: Identifying Immunological Biomarkers for Major Depressive Disorder: Insights From Machine Learning, Single‐Nucleus Bioinformatics, and Experimental Validation
Source: Biomed Res Int. 2026 Apr 11;2026:6184295. doi: 10.1155/bmri/6184295 (PMC13069472; doi:10.1155/bmri/6184295)
Supplement: Supplementary file 1 — Supporting Information Additional supporting information can be found online in the Supporting Information section. Figure S1: This figure illustrates the decision curve analysis (DCA) of the predictive model. Table S1: This table presents the relevant clinical data of patients with severe depression, which is used to analyze the differential genes and common pathways. [file BMRI-2026-6184295-s001.docx]

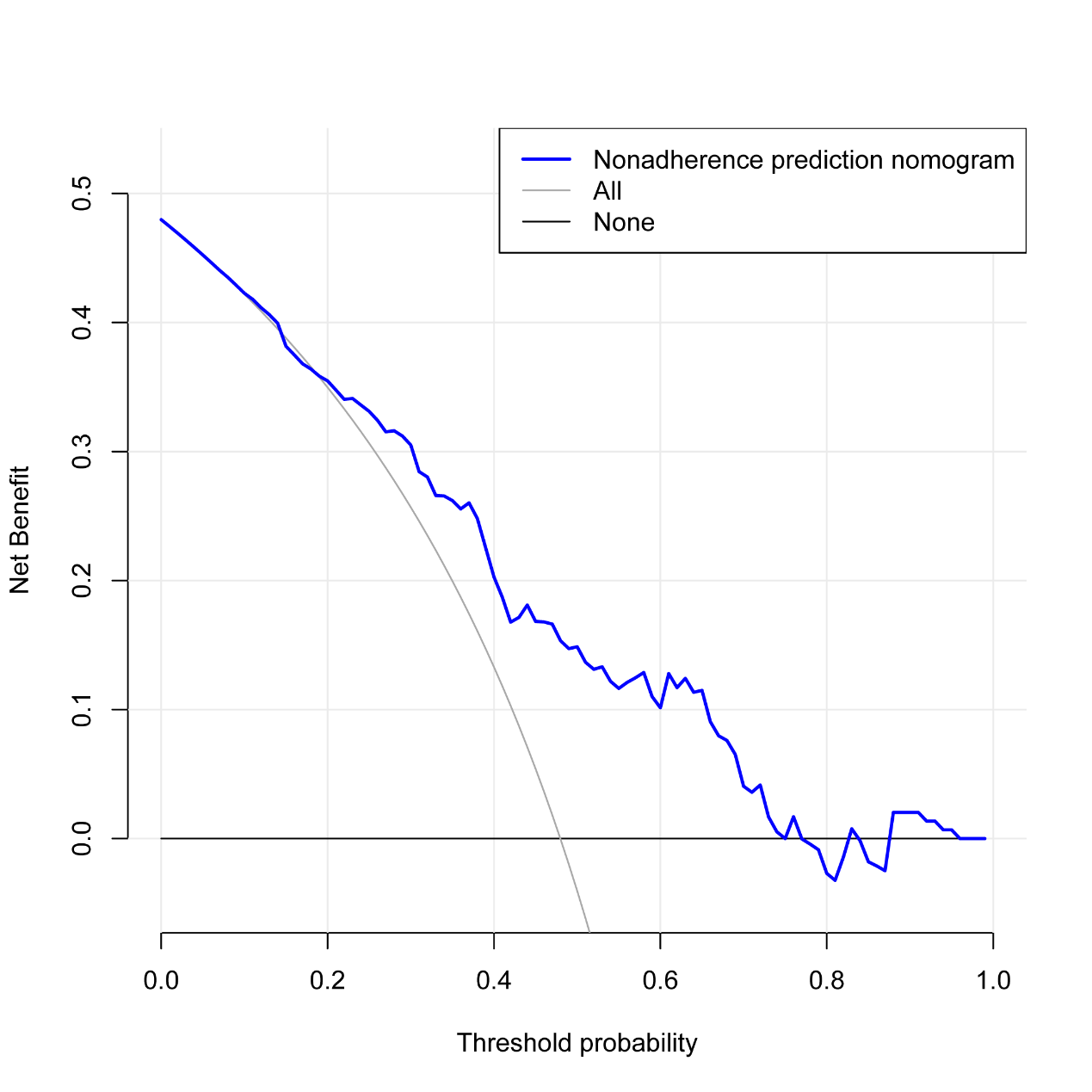


**FigureS1. Decision curve analysis (DCA) of the prediction model.**
Decision curve analysis (DCA) was performed to evaluate the clinical utility of the prediction model across a range of threshold probabilities. The x-axis represents the threshold probability, and the y-axis represents the net benefit. The solid line indicates the proposed prediction model (nomogram), while the dashed lines represent the two extreme strategies of treating all subjects (“All”) or treating none (“None”). The results show that the prediction model yields a higher net benefit than both extreme strategies over a wide range of threshold probabilities, suggesting that the model has potential clinical usefulness and provides stable decision support beyond chance performance.

**Table S1. Clinical data of patients with MDD for analysis of differential genes and common pathways.**

| Characteristics | Total  (N=190) | MDD cohort | Normal cohort | *P*-value |
| --- | --- | --- | --- | --- |
|  |  | (n=79) | (n=111) |  |
| Age (years) | 50.52 ± 12.96 | 48.91 ± 12.82 | 51.67 ± 13.00 | 0.149 |
| Gender |  |  |  | 0.082 |
| Male | 74(38.95) | 25 (31.65) | 49 (44.14) |  |
| Female | 116(61.05) | 54 (68.35) | 62 (55.86) |  |
| Postmortem Interval | 22.00 ± 7.39 | 22.58 ± 7.66 | 21.58 ± 7.19 | 0.358 |
| Tissue ph | 6.69 ± 0.27 | 6.69 ± 0.28 | 6.69 ± 0.26 | 0.908 |
